# MiR-96-5p Induced by Palmitic Acid Suppresses the Myogenic Differentiation of C2C12 Myoblasts by Targeting FHL1

**DOI:** 10.3390/ijms21249445

**Published:** 2020-12-11

**Authors:** Mai Thi Nguyen, Kyung-Ho Min, Wan Lee

**Affiliations:** 1 Department of Biochemistry, Dongguk University College of Medicine, 123 Dongdae-ro, Gyeongju 38066, Korea; nguyenmainhp@gmail.com (M.T.N.); dbdlaeo112@naver.com (K.-H.M.); 2 Channelopathy Research Center (CRC), Dongguk University College of Medicine, 32 Dongguk-ro, Ilsan Dong-gu, 10326 Goyang, Korea

**Keywords:** microRNA, miR-96-5p, FHL1, palmitic acid, differentiation

## Abstract

Skeletal myogenesis is a multi-stage process that includes the cell cycle exit, myogenic transcriptional activation, and morphological changes to form multinucleated myofibers. Recent studies have shown that saturated fatty acids (SFA) and miRNAs play crucial roles in myogenesis and muscle homeostasis. Nevertheless, the target molecules and myogenic regulatory mechanisms of miRNAs are largely unknown, particularly when myogenesis is dysregulated by SFA deposition. This study investigated the critical role played by miR-96-5p on the myogenic differentiation in C2C12 myoblasts. Long-chain SFA palmitic acid (PA) significantly reduced FHL1 expression and inhibited the myogenic differentiation of C2C12 myoblasts but induced miR-96-5p expression. The knockdown of FHL1 by siRNA stimulated cell proliferation and inhibited myogenic differentiation of myoblasts. Interestingly, miR-96-5p suppressed FHL1 expression by directly targeting the 3’UTR of *FHL1* mRNA. The transfection of an miR-96-5p mimic upregulated the expressions of cell cycle-related genes, such as PCNA, CCNB1, and CCND1, and increased myoblast proliferation. Moreover, the miR-96-5p mimic inhibited the expressions of myogenic factors, such as myoblast determination protein (MyoD), myogenin (MyoG), myocyte enhancer factor 2C (MEF2C), and myosin heavy chain (MyHC), and dramatically impeded differentiation and fusion of myoblasts. Overall, this study highlights the role of miR-96-5p in myogenesis via FHL1 suppression and suggests a novel regulatory mechanism for myogenesis mediated by miRNA in a background of obesity.

## 1. Introduction

Skeletal muscle is the most abundant tissue in the human body and plays an essential role in a wide range of functions, including metabolism, respiration, and locomotion [1]. The maintenance of skeletal muscle mass and integrity by myogenesis is essential for whole-body homeostasis and the proper physical and metabolic functioning of skeletal muscle [2]. On the other hand, dysregulation of myogenesis is causally linked to the development of sarcopenia, known as the age-related loss of skeletal muscle, as well as diverse pathological conditions, including metabolic myopathy and muscular dystrophy [3,4]. Skeletal myogenesis is a highly coordinated, complex process involving satellite cell activation, myoblast proliferation, cell cycle exit, and myoblast fusion into multinucleated myofibers [2]. During myogenic progression, the transcriptional activation of muscle-specific genes is mainly regulated by myogenic regulatory factors (MRFs), such as myogenic factor 5 (Myf5), myogenic differentiation (MyoD), myogenic regulatory factor 4 (MRF4), and myogenin (MyoG). In addition, the myocyte-specific enhancer factor 2 (MEF2) family of transcription factors, which promote the myogenic differentiation of progenitor cells into myotubes, are also important regulators of skeletal muscle differentiation [5]. Accumulating evidence suggests that excessive intake of saturated fatty acids (SFA) increases intramuscular fat deposition and results in lipotoxicity linked to mitochondrial dysfunction, apoptosis, and inflammation, which eventually leads to muscle atrophy and wasting [6,7]. Furthermore, recent studies have revealed that SFA can inhibit the activation of MRFs and impede the myogenic differentiation in diverse progenitor cells [8,9,10,11]. However, the underlying mechanisms responsible for the inhibition of myogenic differentiation by SFA in myoblasts remain substantially unknown.

MicroRNAs (miRNAs) are a large family of endogenously expressed small non-coding RNAs of approximately 19 to 22 nucleotides that function in gene expression as negative regulators by binding to the 3’UTRs of target mRNAs [12]. Although the targets and functions of miRNAs have not been fully understood, evidence suggests that they are widely involved in normal and pathophysiological cellular processes [12]. Therefore, dysregulation of miRNA expression is closely associated with many diseases, including cancer, neurodegeneration, and metabolic diseases [13]. In recent decades, a growing body of research has expanded our understanding of the essential roles miRNAs play in myogenesis and their impacts on muscle homeostasis, muscle mass maintenance, and the pathogeneses of myopathies [14,15]. Many studies have shown that miRNAs are dysregulated in obesity and suggested that exosomal miRNAs might represent a new class of endocrine factors [16,17]. However, the mechanism whereby miRNAs dysregulated by SFA or obesity are causally connected to impaired myogenic differentiation remains poorly understood.

Four-and-a-half LIM domains protein 1 (FHL1) is encoded by the *FHL1* gene and is a member of the FHL family of proteins involved in nuclear–cytoplasmic networking, muscle development, and sarcomere assembly [18]. Since FHL1 is highly expressed in skeletal muscle, an increasing number of studies have focused on the role of FHL1 in skeletal muscle integrity and myopathy [19], and several recent studies have demonstrated that FHL1 is a crucial regulator of skeletal myogenesis and muscle maintenance [20,21,22,23]. Mutations in the *FHL1* gene have been reported to be associated with various human myopathies, including X-linked myopathy with postural muscle atrophy [24], reducing body myopathy [25], scapuloperoneal myopathy [26], and Emery–Dreifuss muscular dystrophy [27]. Interestingly, FHL1-transgenic mice displayed skeletal muscle hypertrophy and a fiber-type transition, resulting in increased whole-body strength and fatigue resistance [20]. Moreover, FHL1 overexpression increased myotube fusion and caused myotube hypertrophy in C2C12 cells, indicating that it has the potential to enhance skeletal myogenesis and muscle mass [20,21]. FHL1 knockout in mice, on the other hand, increased skeletal muscle autophagic activity and induced myopathies with irregular structure and muscle fiber size, suggesting an important role of FHL1 in myogenesis and muscle integrity [21,22,23]. However, the association between FHL1 expression and SFA accumulation in myocytes has not been explored. It is still unclear if FHL1 expression is suppressed by particular miRNAs induced by SFAs and how they influence myogenic differentiation.

In this study, we revealed the roles of miR-96-5p on the myogenic differentiation in C2C12 myoblasts. Treatment with palmitic acid (PA), the most enriched SFA in diet, on C2C12 myoblasts suppressed FHL1 expression and myogenic differentiation, but induced miR-96-5p expression. The depletion of FHL1 increased cell proliferation and impaired myogenic differentiation of myoblasts. Interestingly, miR-96-5p mimic reduced FHL1 expression by directly targeting the 3’UTR of *FHL1* mRNA. Moreover, miR-96-5p mimic enhanced the proliferation of myoblasts, inhibited the expressions of myogenic factors, and consequently inhibited myogenic differentiation in C2C12 myoblasts. Therefore, this study highlights the crucial role of miR-96-5p in myogenic differentiation through FHL1 suppression and suggests a novel miRNA-mediated myogenic regulatory mechanism in a background of obesity.

## 2. Results

### 2.1. PA Inhibited Myogenic Differentiation and FHL1 Expression but Induced miR-96-5p Expression

**Figure 1:**
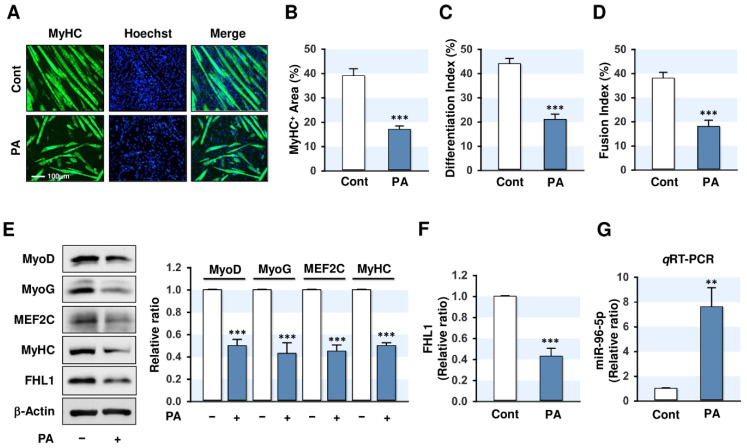
Palmitic acid (PA) impairs myogenic differentiation and upregulates miR-96-5p in C2C12 myoblasts. C2C12 myoblasts were pretreated with PA (100 μM) for 24 h and differentiated up to five days. (**A**) Immunofluorescence staining with a specific antibody against MyHC (green). Hoechst (blue) was used to stain nuclei. Scale bar: 100 μm. (**B**–**D**) MyHC-positive area, differentiation index, and fusion index were determined as described in Materials and Methods. (**E**) Expressions of myogenic factors and FHL1. (**F**) Quantitative analysis of FHL1 expression. (**G**) *q*RT-PCR of miR-96-5p (24 h after PA treatment) following normalization with U6 control. Expression levels in immunoblots were normalized with β-Actin. The values are expressed as the relative ratio, where the intensity of day 0 was set to one. Results are expressed as means ± SEMs (*n* > 3). **, *p* < 0.01; ***, *p* < 0.001 vs. BSA control.

To determine the effect of PA on myogenic differentiation, the cell viability of C2C12 myoblasts was analyzed at various concentrations of PA, as shown in (Supplementary File Figure S1). Since less than 100 μM of PA had no cytotoxic effects in C2C12 myoblasts, cells were treated with PA (100 μM) for 24 h before differentiation. Myogenic differentiation was then assessed until day five based on the myotube formation and expressions of myogenic factors, such as MyoD, MyoG, MEF2C, and MyHC. Immunocytochemical analysis showed that PA drastically reduced the MyHC-positive area, differentiation index, and fusion index (Figure 1A–D), suggesting impaired myogenic differentiation of progenitor cells by PA. Subsequently, the expressions of myogenic factors were determined by immunoblotting in control and PA-treated myoblasts. As shown in Figure 1E, the expressions of MyoD, MyoG, MEF2C, and MyHC were significantly reduced by PA, indicating that inhibition of the myogenic differentiation by PA was linked to the suppression of myogenic factors, which is known to play essential roles in the differentiation, fusion, and maturation of progenitor cells.

Next, we examined whether PA suppresses FHL1 expression in C2C12 myoblasts since FHL1 was previously reported to be involved in skeletal muscle maintenance and myogenesis [20,21,22,23]. As shown in Figure 1E,F, PA reduced FHL1 expression in myoblasts drastically, implying that FHL1 downregulation by PA was associated with the suppression of myogenic factors and myogenic differentiation. To determine how PA suppressed FHL1 expression, we investigated whether miRNAs induced by PA might control FHL1 expression and myogenic differentiation in myoblasts. According to the miRNA microarray analysis in PA-treated myoblasts, many miRNAs are upregulated by PA (data not shown). Among those, miR-96-5p was selected for further experiments because, as in silico analysis predicted, it targets *FHL1* 3’UTR (Figure 3A). The *q*RT-PCR analysis confirmed our microarray findings by showing PA-treated C2C12 myoblasts displayed a more than seven-fold increase in miR-96-5p expression (Figure 1G). Collectively, PA was found to suppress FHL1 expression, downregulate myogenic factors, and inhibit the differentiation, but induce miR-96-5p expression in C2C12 myoblasts.

### 2.2. FHL1 Is Essential for Myogenic Differentiation

**Figure 2:**
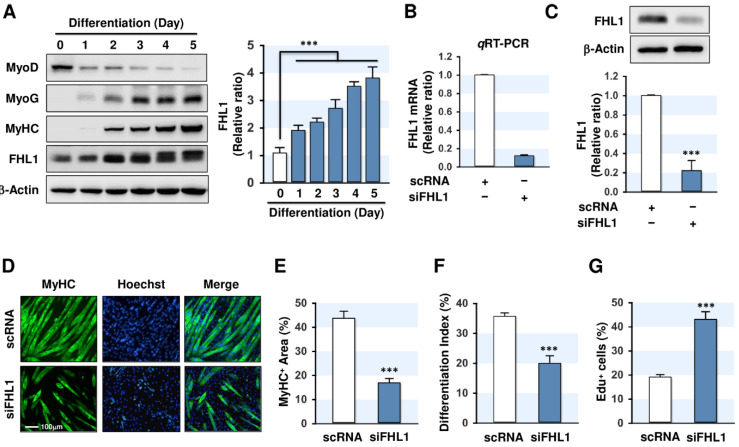
Knockdown of FHL1 impairs myogenic differentiation. (**A**) C2C12 myoblasts were differentiated up to five days, and the expression of FHL1 was analyzed. Expression of FHL1 in immunoblots was normalized with β-Actin. The values are expressed as the relative ratio, where the intensity of day 0 was set to one. (**B**) *q*RT-PCR analysis of FHL1 expression after 24 h of transfection with 100 nM of scRNA control or FHL1 siRNA (siFHL1). GAPDH was used as a control. (**C**) Immunoblot analysis of FHL1 after 24 h of transfection. (**D**) C2C12 myoblasts were transfected with 100 nM of scRNA control or siFHL1 and were differentiated for five days. Immunofluorescence staining with a specific antibody against MyHC (green). Hoechst (blue) was used to stain nuclei. Scale bar: 100 μm. (**E**–**G**) MyHC-positive area, differentiation index, and Edu-positive cells were determined as described in Materials and Methods. Results are expressed as means ± SEMs (*n* > 3). ***, *p* < 0.001.

Although FHL1 is suggested as an important factor of skeletal muscle maintenance and myogenesis [20,21,22,23], the roles and regulation of FHL1 during myogenic differentiation are currently unknown. To determine the expression profile of FHL1 during myogenic differentiation, we differentiated C2C12 myoblasts for up to five days, and then, the expression of FHL1 and myogenic factors were analyzed. As shown in Figure 2A, the cellular level of MyoD was gradually decreased during myogenic differentiation, whereas the expressions of MyoG and MyHC were substantially increased. Interestingly, the level of FHL1 was upregulated significantly as compared to the level of undifferentiated myoblasts (Figure 2A). Therefore, we examined whether FHL1 reduction inhibits the myogenic differentiation in C2C12 myoblasts. Transfection of siRNA against FHL1 (siFHL1, 100 nM), which was used to knockdown FHL1, successfully suppressed the mRNA and protein levels of FHL1 by approximately 80–90% (Figure 2B,C). This effect was sustained during the differentiation period (Figure S2). Under this condition, the knockdown of FHL1 by siFHL1 drastically inhibited the myotube formation of C2C12 myoblasts (Figure 2D). Quantitative analysis, namely MyHC-positive area and differentiation index, clearly suggested that the knockdown of FHL1 resulted in impaired myogenic differentiation of C2C12 myoblasts (Figure 2E,F). As myoblast proliferation and differentiation into myotubes exhibit an inverse relationship during myogenesis [2], we hypothesize that FHL1 may modulate the proliferation of myoblasts, and thus, regulates myogenic factors and differentiation. To investigate the role of FHL1 in cell proliferation, we investigated ethynyl deoxyuridine (EdU) incorporation after transfecting C2C12 myoblasts with scRNA or FHL1 siRNA. Interestingly, FHL1 siRNA significantly increased the percentage of EdU-positive cells compared with scRNA-transfected cells (Figure 2G), indicating that FHL1 knockdown enhanced myoblast proliferation. Thus, these results suggest that the expression of FHL1 is indispensable for myogenic differentiation and myotube formation in C2C12 myoblasts.

### 2.3. MiR-96-5p Directly Targeted the 3’UTR of FHL1

**Figure 3:**
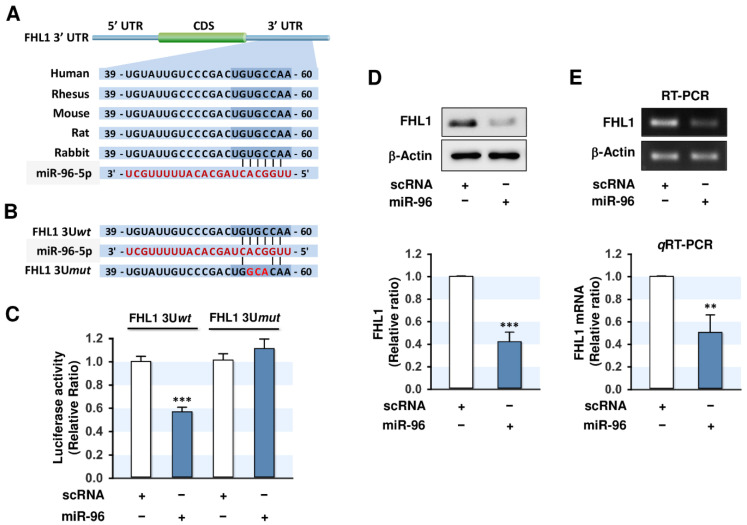
MiR-96-5p directly targets the 3’UTR of *FHL1* and reduces FHL1 expression. (**A**) Sequence alignment of the 3’UTR fragments of *FHL1* with miR-96-5p. (**B**) Sequences of the wild-type (FHL1 3U*wt*) and mutant (FHL1 3U*mut*) of *FHL1* 3’UTRs. (**C**) Luciferase reporter assays using scRNA or miR-96-5p cotransfected with a segment of wild-type (FHL1 3U*wt*) or mutant (FHL1 3U*mut*) *FHL1* 3’UTR in pmirGLO vectors. (**D**) Immunoblot analysis of FHL1 at 24 h after transfection with the 100 nM of scRNA control or miR-96-5p mimic. (**E**) RT-PCR (upper) and *q*RT-PCR (lower) analysis of FHL1 expression at 24 h after transfection with 100 nM of scRNA control or miR-96-5p mimic. The level of expression was normalized to the amount of β-Actin. The values are expressed as the relative ratio, where the intensity of normalized scRNA control was set to one. Results are expressed as means ± SEMs (*n* > 3). **, *p* < 0.01; ***, *p* < 0.001 vs. scRNA.

Since the expression of FHL1 was found to be inversely related to the level of miR-96-5p in PA-treated myoblasts (Figure 1F,G), we next examined whether miR-96-5p directly regulates FHL1 expression. In silico miRNA target prediction analysis, such as TargetScan and miRWalk, showed that FHL1 is a possible target of miR-96-5p because the 3′UTR of *FHL1* includes a tentative binding site for the seed sequence of miR-96-5p (Figure 3A). To confirm direct targeting of *FHL1* 3’UTR by miR-96-5p, the luciferase reporter constructs with a 3’UTR segment of *FHL1* containing either a tentative binding site of miR-96-5p (wild-type; FHL1 3U*wt*) or mutated three nucleotides of the seed sequences (FHL1 3U*mut*) were cloned into the pmirGLO vector (Figure 3B). Subsequently, C2C12 cells were cotransfected with the pmirGLO vector and either the mature miR-96-5p mimic or scRNA control. As shown in Figure 3C, the transfection of the miR-96-5p mimic reduced luciferase activity of the reporter containing a wild-type (FHL1 3U*wt*) compared to the scRNA control. On the other hand, mutations in the tentative miR-183-5p binding site on the *FHL1* 3’UTR (FHL1 3U*mut*) almost completely abolished the inhibitory effect of miR-96-5p on luciferase activity observed in FHL1 3U*wt*. As direct binding of miR-96-5p to the 3’UTR of *FHL1* was confirmed by luciferase reporter analysis, miR-96-5p induction might inhibit FHL1 expression in myoblasts. To determine this further, we next transfected C2C12 myoblasts with scRNA or miR-96-5p mimic and then FHL1 expression was analyzed. As expected, the transfection of miR-96-5p mimic decreased the protein level of FHL1 in C2C12 myoblasts significantly compared to the scRNA control (Figure 3D). Furthermore, the mRNA levels of *FHL1* were also suppressed by the transfection of the miR-96-5p mimic according to RT-PCR and *q*RT-PCR analysis (Figure 3E). These results show that miR-96-5p negatively regulates the expression of FHL1 through direct targeting to *FHL1* 3’UTR.

### 2.4. MiR-96-5p Increased the Proliferation of C2C12 Myoblasts

**Figure 4:**
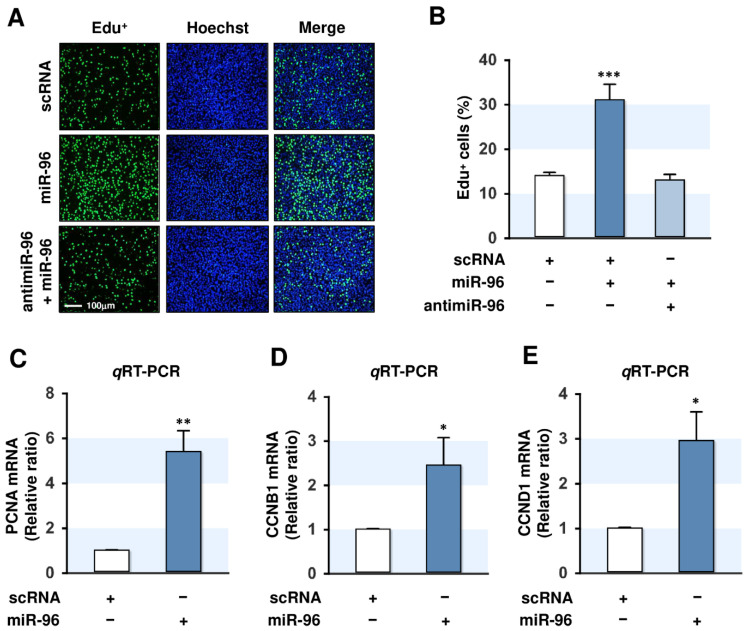
MiR-96-5p stimulates the proliferation of C2C12 myoblasts. C2C12 myoblasts were transfected with 100 nM of scRNA control, miR-96-5p mimic, or antimiR-96-5p. (**A**) After 24 h of transfection, the cells were labeled with 10 µM of EdU (green) for 4 h, and nuclei were stained with Hoechst (blue). (**B**) Percentages of EdU-positive cells were analyzed by ImageJ software. (**C**–**E**) *q*RT-PCR analysis of PCNA, CCNB1, and CCND1 expression at 24 h after transfection with 100 nM of scRNA control or miR-96-5p mimic. The level of expression was normalized to the amount of U6. The values are expressed as the relative ratio, where the intensity of normalized scRNA control was set to one. Results are expressed as means ± SEMs (*n* > 3). *, *p* < 0.05; **, *p* < 0.01; ***, *p* < 0.001 vs. scRNA.

**Figure 5:**
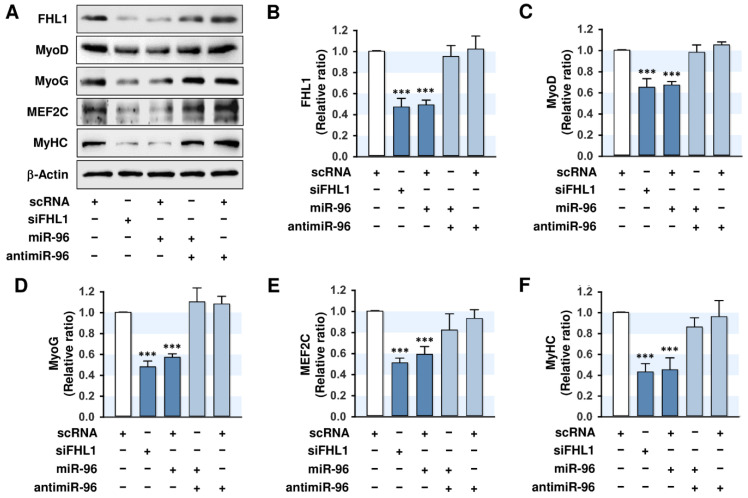
MiR-96-5p suppresses the expressions of myogenic factors and FHL1. C2C12 myoblasts were transfected with 100 nM of scRNA control, siFHL1, miR-96-5p mimic, or antimiR-96-5p. (**A**) Representative immunoblots obtained after differentiation for three days. (**B**) Quantitative analysis of FHL1 expression. (**C**–**F**) Quantitative analysis of MyoD, MyoD, MEF2C, and MyHC expressions. Expression levels were normalized vs. β-Actin. The values are expressed as the relative ratio, where the intensity of normalized scRNA control was set to one. Results are expressed as means ± SEMs (*n* > 3). ***, *p* < 0.001 vs. scRNA.

The differentiation of myoblasts to myotubes requires proliferation arrest and myogenic factor activation [28]. Therefore, we examined the role played by miR-96-5p in the regulation of myoblast proliferation 24 h of transfection with scRNA, miR-96-5p mimic, or antimiR-96-5p (an inhibitor of miR-96-5p) in C2C12 myoblasts. According to *q*RT-PCR analysis, transfection of miR-96-5p mimic (100 nM) markedly increased (>100-fold) the cellular level of miR-96-5p, whereas antimiR-96-5p transfection (100 nM) suppressed endogenous miR-96-5p level by 40% (data not shown). Under this experimental condition, the transfection of miR-96-5p mimic significantly increased EdU-positive myoblasts compared with scRNA-transfected cells, whereas antimiR-96-5p completely blocked the effect of miR-96-5p on EdU incorporation in myoblasts (Figure 4A,B), indicating that miR-96-5p mimic enhanced myoblast proliferation. The *q*RT-PCR analysis showed that proliferating cell nuclear antigen (PCNA), which is indispensable for cell proliferation, was dramatically upregulated in myoblasts transfected with miR-96-5p mimic (Figure 4C). To confirm the enhancement of proliferation, the expressions of cyclins (CCNB1 and CCND1, mediators of cell-cycle progression) were analyzed as markers of cell proliferation. CCNB1 and CCND1 expressions were significantly upregulated by miR-96-5p mimic (Figure 5D,E). Taken together, these results suggest that miR-96-5p positively regulates the proliferation of C2C12 myoblasts.

### 2.5. MiR-96-5p Inhibited the Expressions of Myogenic Factors

Because miR-96-5p mimic increased cell proliferation (Figure 4), we next investigated whether the induction of miR-96-5p also suppresses the expressions of myogenic factors. C2C12 myoblasts transfected with scRNA, siFHL1, miR-96-5p mimic, or antimiR-96-5p were cultured in a differentiation medium for three days, and then the protein levels of MyoD, MyoG, MEF2C, and MyHC were analyzed. Transfection of siFHL1 reduced the level of FHL1 by about 60–70% vs. scRNA control levels (Figure 5A,B), and the knockdown of FHL1 by siFHL1 significantly suppressed the expressions of myogenic factors, such as MyoD, MyoG, and MEF2C in C2C12 myoblasts (Figure 5A,C–F). Interestingly, the transfection of miR-96-5p mimic decreased the expression of FHL1 drastically and inhibited the expressions of myogenic factors, such as MyoD, MyoG, and MEF2C, vs. scRNA-transfected controls (Figure 5A,C–F). MyHC expression as a terminal myogenic differentiation marker was reduced by either siFHL1 or miR-96-5p, which suggested that FHL1 suppression or miR-96-5p induction are causally linked to the inhibition of myogenic factor expression. Furthermore, cotransfection with antimiR-96 almost completely restored the inhibitory effect of miR-96-5p on the expressions of FHL1 and myogenic factors (Figure 5A–F). Therefore, these findings suggest that the upregulation of miR-96-5p resulted in the downregulation of FHL1, which is essential for the expression of myogenic factors, and consequently, this upregulation suppressed the expressions of MyoD, MyoG, MEF2C, and MyHC in C2C12 myoblasts.

### 2.6. MiR-96-5p Inhibited the Myogenic Differentiation of C2C12 Myoblasts

**Figure 6:**
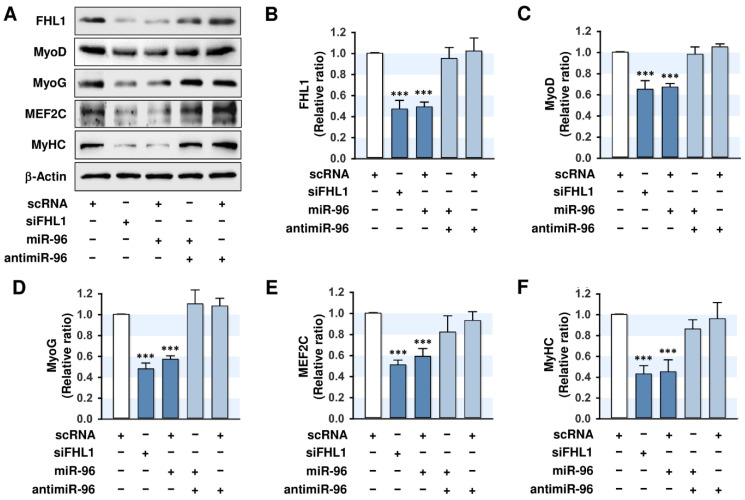
MiR-96-5p impairs myogenic differentiation. C2C12 myoblasts were transfected with 100 nM of scRNA control, siFHL1, miR-96-5p mimic, or antimiR-96-5p. (**A**) Immunofluorescence staining with a specific antibody against MyHC (green). Hoechst (blue) was used to stain nuclei. Scale bar: 100 μm. MyHC-positive area (**B**), differentiation index (**C**), fusion index (**D**), and myotube width (**E**) were determined as described in Materials and Methods. Results are expressed as means ± SEMs (*n* > 3). ***, *p* < 0.001 vs. scRNA.

Since miR-96-5p was found to increase myoblast proliferation and suppress the expression of myogenic factors, we examined whether miR-96-5p inhibits myogenic differentiation. C2C12 myoblasts were transfected with scRNA, siFHL1, miR-96-5p mimic, or antimiR-96-5p, and cultured in a differentiation medium for five days (Figure 6). Myogenic differentiation was assessed by immunocytochemistry using MyHC antibody and Hoechst and subsequently subjected to quantitative analysis. The knockdown of FHL1 using siFHL1 drastically inhibited myotube formation in C2C12 myoblasts assessed by MyHC immunofluorescence (Figure 6A). MyHC-positive area, differentiation index, fusion index, and myotube width showed that knockdown of FHL1 was causally linked to the impaired myogenic differentiation of myoblasts (Figure 6B–E). As expected, the transfection of miR-96-5p mimic instead of siFHL1 impaired the differentiation of C2C12 myoblasts as determined by immunocytochemistry and quantitative analysis of differentiation (Figure 6). Moreover, cotransfection with antimiR-96-5p almost entirely blocked the miR-96-5p mimic-induced inhibitions of differentiation and myotube formation (Figure 6A–E). Thus, these results suggest that miR-96-5p suppresses the myogenic differentiation and myotube formation of C2C12 myoblasts.

## 3. Discussion

Despite growing interest in the roles of miRNAs on myogenesis and muscle homeostasis, the targets and myogenic regulatory mechanisms of miRNAs are largely unknown. This study unveils the critical role played by miR-96-5p on the myogenic differentiation in C2C12 myoblasts. The key contributions made by this study to current knowledge are as follows: (i) PA inhibited FHL1 expression and suppressed the myogenic differentiation of myoblasts. (ii) PA upregulated miR-96-5p expression in myoblasts. (iii) The knockdown of FHL1 inhibited the expressions of myogenic factors and differentiation. (iv) miR-96-5p targeted the 3’UTR of *FHL1* directly and thereby downregulated FHL1 expression. (v) Transfection of miR-96-5p mimic increased myoblast proliferation, suppressed the expressions of myogenic factors, and impaired the myogenic differentiation of myoblasts. Overall, our findings highlight the potential role of miR-96-5p in myogenesis via the repression of FHL1 and provide evidence of miRNA-mediated myogenic regulation in an association with SFA.

The accumulation of SFA in skeletal muscle is known to trigger lipotoxicity, as manifested by mitochondrial dysfunction, inflammation, and apoptosis, and eventually lead to muscle atrophy and wasting [6,7]. Moreover, recent studies indicate that PA might inhibit the expressions of myogenic regulatory factors and the differentiation of various cells, including primary skeletal muscle satellite cells [11] and C2 myoblasts [10]. In the present study, we found that PA suppressed the myogenic differentiation of C2C12 myoblasts accompanied by dramatic reductions in myogenic factors, such as MyoD, MyoG, and MEF2C (Figure 1). Furthermore, we found for the first time that PA inhibited FHL1 expression significantly in C2C12 myoblasts (Figure 1). Defects in the *FHL1* gene are known to be linked to multiple human myopathies [24,25,26,27], but little is known of the functions and regulation of FHL1 in skeletal muscle. Nevertheless, some evidence suggests that FHL1 plays an essential role in myogenesis and skeletal muscle homeostasis. In genetically modified mouse models, FHL1 knockout resulted in muscle fiber abnormalities, reduced muscle strength, and activated autophagy in skeletal muscle [22,23]. On the other hand, FHL1-transgenic mice exhibited whole-body fatigue resistance with hypertrophy in skeletal muscle [20]. Mechanistically, knockdown of FHL1 reduced myotube formation in chicken primary myoblasts by inhibiting myogenic regulatory factors, such as MyoD and MyoG [29]. In addition, knockdown of FHL1 upregulated the expressions of Atrogin-1 and MuRF1, which cause muscle atrophy and wasting [29]. In the present study, knockdown of FHL1 with siRNA decreased the expressions of MyoD, MyoG, MEF2C, and MyHC, and inhibited myotube formation in C2C12 cells (Figure 5 and Figure 6), which is in-line with observations in primary chicken myoblasts [29]. Given that PA drastically suppressed FHL1 expression and simultaneously inhibited the expressions of myogenic factors and myotube formation in C2C12 myoblasts, we suggest that the suppression of FHL1 is a crucial contributor to the impairment of myogenic differentiation by PA.

Increasing evidence suggests that miRNAs are important regulators of myogenesis and muscle homeostasis [13,14]. This study reveals the novel roles played by miR-96-5p on FHL1 expression and myogenic differentiation in C2C12 myoblasts and confirms our hypothesis that PA-induced miRNAs impair myogenic differentiation by targeting FHL1. Hsa-miR-96-5p is a member of the miR-183 family, which consists of three highly conserved miRNAs (miR-96, miR-182, and miR-183) in vertebrates [30]. According to recent findings, this family is involved in a wide range of normal physiological processes, such as cell proliferation, apoptosis, immunity, and metabolism [30,31,32]. Although miR-96-5p is ubiquitously expressed in various tissues, including muscle, liver, and adipose tissues [33], previous studies on the role and biological significance of miR-96-5p have focused mainly on oncogenesis and cancer progression [32,34,35]. As a result, miR-96-5p is considered an oncomiR that facilitates the development of malignancies by promoting cancer cell growth, proliferation, and survival [36,37]. However, the roles of miR-96-5p in myogenesis have not been previously investigated. The results of this study provide evidence of miR-96-5p-mediated myogenic regulation, and that miR-96-5p inhibits FHL1 expression by directly binding to the 3’UTR of *FHL1*. Since the silencing of FHL1 is causally linked to the inhibition of myogenic differentiation and there are no tentative binding sites for miR-96-5p on the 3’UTRs of other myogenic factors, such as MyoD, MyoG, MEF2C, and MyHC, the impairment of myogenic differentiation observed in miR-96-5p-transfected myoblasts can be primarily attributed to the repression of FHL1 by miR-96-5p. Therefore, the induction of miR-96-5p might be a detrimental factor for myogenesis and muscle mass maintenance by reducing FHL1 expression.

This study revealed that miR-96-5p mimic increased myoblast proliferation in myoblasts (Figure 4). Myoblast proliferation and differentiation exhibit an opposite association during myogenesis [2]. Therefore, proliferation arrest and cell cycle exit are typically necessary to differentiate from myoblasts to myotubes [28]. Previously, FHL1 has been reported to function as a tumor suppressor with inhibitory effects on cell growth, proliferation, and migration in various cancer cells [38]. FHL1 induced the expression of tumor suppressor genes by increasing the nuclear translocation of Smad4 in hepatocellular carcinoma cells [39] and inhibiting the phosphorylation of AKT in human breast cancer cells [40]. Furthermore, FHL1 suppressed the levels of G1 and G2/M phase-related proteins, including cyclin A, B1, D, and E, resulting in G1 and G2/M cell cycle arrest in lung cancer cells [41]. On the other hand, the downregulation of FHL1 has been detected in multiple gastric and colon cancer, and the silencing of FHL1 promoted cell growth, migration, and invasion in gastrointestinal cancer cells [42]. In this study, we found that miR-96-5p acts as a negative regulator of FHL1 and knockdown of FHL1 by miR-96-5p caused myoblast proliferation by regulating the expression of PCNA, CCNB1, and CCND1, which promote cell cycle progression [43,44,45]. Thus, it is suggested that knockdown of FHL1 by miR-96-5p in C2C12 myoblasts upregulates cycle progression-related genes, which lead to myoblast proliferation, and consequently impaired myogenic differentiation.

The underlying regulatory molecular mechanism whereby PA induces miR-96-5p expression in myoblasts is undetermined in this study. However, certain transcription factors activated by PA or obesity may trigger the transcriptional activation of miR-96-5p. According to in silico analysis of transcription factor binding sites, the promoter regions of miR-96-5p contain putative binding sites for various transcription factors associated with adipogenesis and obesity, such as SREBPs, PPARγ, and C/EBPα. SREBPs play critical roles in the regulation of fatty acid metabolism and adipogenesis, and their activations are linked to a high-fat diet and obesity [46]. Since the promoter of miR-96-5p is directly targeted by SREBPs [47], the mechanism of miR-96-p induction in myoblasts treated with PA may be associated with SREBP activation. PPARγ is an important transcription factor in mammalian adipogenesis and is closely related to obesity [48]. The upregulation of miR-96-5p by PPARγ was previously reported in differentiating 3T3-L1 adipocytes [49]. Moreover, PPARγ is also coactivated with C/EBPα, an adipogenic transcription factor [50], which is also activated in high-fat diet-induced obese mice [51]. Thus, it is proposed that the activation of PPARγ and C/EBPα may contribute to the upregulation of miR-96-5p by PA. Although the induction of miR-96-5p has been reported in non-muscle tissues, miR-96-5p was recently suggested to play a role as a plasma-driven exosomal miRNA in inter-tissue communication [52,53]. Further research is needed to determine how transcriptional factors regulate miR-96-5p. Nonetheless, results available to date indicate that miR-96-5p may be a novel key player in the association between obesity and muscle mass reduction.

## 4. Materials and Methods

### 4.1. Cell Culture, Differentiation, and PA Treatment

C2C12 myoblasts, a murine-derived muscle cell line (ATCC, Manassas, VA, USA), were maintained in a growth medium (GM) consisting of Dulbecco’s modified Eagle’s medium (DMEM) supplemented with 10% fetal bovine serum (FBS) and 1% penicillin-streptomycin (Gibco, Thermo Fisher Scientific, Waltham, MA, USA) in a humidified incubator (Thermo Fisher Scientific) containing 5% CO_2_ at 37 °C. Cells were seeded in 6-well plates at a density of 1.3 × 10^5^ cells/well and incubated for 24 h before transfection. After reaching 80–90% confluence, the cells were switched from 10% FBS to 2% horse serum (Gibco) to induce myogenic differentiation. When necessary, cells were treated with BSA-conjugated PA for 24 h in GM before differentiation. Cytotoxicity at a given concentration of PA was assessed using a Quanti-Max Cell Viability Assay Kit (Biomax, Seoul, Korea) according to the manufacturer’s instructions.

### 4.2. Transfection of Oligonucleotides

C2C12 myoblasts in 6-well dishes or 35 mm dishes were transfected with 100 nM of scrambled control RNA (scRNA), FHL1 siRNA (siFHL1), miR-96-5p mimic, or antimiR-96-5p (an inhibitor of miR-96-5p; a 2′-O-methyl-modified antisense oligonucleotide against mature miR-96-5p) (Genolution, Seoul, Korea) using Lipofectamine 2000 (Invitrogen, Carlsbad, CA, USA) according to the manufacturer’s protocol. The sequences of oligonucleotides are listed in Table 1.

### 4.3. RNA Preparation and Quantitative Reverse Transcription-Polymerase Chain Reaction (qRT-PCR)

Total RNA was isolated from C2C12 cells using Qiazol (Qiagen, Hilden, Germany) and further purified with a miRNeasy Mini Kit (Qiagen), following the manufacturer’s instructions. cDNAs were synthesized using a miScript II RT Kit (Qiagen) according to the manufacturer’s protocol. To determine mRNA and miRNA expressions, *q*RT-PCR was carried out using SYBR Green I and iTaq polymerase (Promega, Madison, WI, USA) in a LightCycler 480 (Roche Applied Science, Penzberg, Germany). The primers used for RT-PCR or *q*RT-PCR and reaction conditions are listed in Table 1. Relative expression levels of indicated genes were calculated using the 2^−ΔΔCt^ method. Results were normalized to GAPDH or U6 expression.

### 4.4. Dual-Luciferase Reporter Assay

Using the primer sets described in Table 1, murine *FHL1* 3’UTR (252 nt) was amplified by RT-PCR and subcloned into pmirGLO (Promega). MiR-96-5p binding sites on the *FHL1* 3’UTR were mutated by site-directed mutagenesis according to the manufacturer’s protocol (Promega). Cells (5 × 10^4^) were seeded into 12-well dishes. The day after, cells were cotransfected with scRNA or miR-96-5p mimic and pmirGLO luciferase vector containing either the *FHL1* 3’UTR wild-type or mutant sequence using Lipofectamine 2000. Dual-luciferase reporter gene assays were performed 24 h after transfection, as described previously [54].

### 4.5. Immunoblot Analysis

C2C12 cells were homogenized using a lysis buffer containing a phosphatase inhibitor cocktail, and lysates were dissolved in Laemmli solution, as previously described [55]. Total protein concentration was measured with a BSA standard line. Proteins were separated by SDS-PAGE and transferred to nitrocellulose membranes. The membranes were blocked with 5% skim milk (Becton, France) for 1 h. Afterward, immunoblotting was conducted using specific antibodies described in Table 2. All immunoblots were visualized using a Femto reagent (Thermo Fisher Scientific) and quantified by densitometry using an analytical scanning system (Fusion Solo, Paris, France).

### 4.6. Immunofluorescence Analysis

Five days after transfection with scRNA, siFHL1, miR-96-5p mimic, or antimiR-96-5p, C2C12 cells were washed three times with phosphate-buffered saline solution (PBS), fixed in 4% paraformaldehyde for 10 min, permeabilized with 0.3% Triton X-100 at 37 °C for 15 min, and blocked with 3% bovine serum albumin (BSA) (GenDEPOT, Barker, TX, USA) solution for 2 h in PBS. All steps were performed at room temperature. Cells were then incubated with primary antibodies (MyHC, 1: 100 dilution) at 4 °C overnight, and this was followed by incubation with Alexa 488-conjugated secondary antibody (Thermo Fisher Scientific) at room temperature for another 1.5 h. Nuclei were stained with Hoechst for 15 min. Finally, cells were photographed under a fluorescence microscope (Leica, Wetzlar, Germany). For quantitative analysis, differentiation indices were calculated by expressing numbers of nuclei in MyHC-positive myotubes as percentages of total numbers of nuclei, and fusion indices were calculated by expressing numbers of myotubes with three or more nuclei as percentages of the total numbers of nuclei. The number of myotubes, myotube widths, and MyHC-positive areas were measured using Image J Software. All of the data were collected from at least three independent cultures using at least five randomly selected areas/culture.

### 4.7. Ethynyl Deoxyuridine (EdU) Assay

EdU assays were performed to measure cell proliferation using a Click-iT EdU Cell Proliferation Kit (Invitrogen). Briefly, after 24 h of transfection, C2C12 myoblasts were labeled with 10 µM of EdU for 4 h, fixed with 4% paraformaldehyde for 10 min, and permeabilized with 0.3% Triton X-100 in PBS for 30 min. Cells were then incubated with 0.3 mL of Click-iT reaction cocktail for 20 min, and nuclei were stained with Hoechst for 15 min. All images were taken using an optical microscope (Leica). Percentages of EdU-positive cells and total numbers of nuclei were then determined. Images were collected from at least three independent experiments using at least five randomly selected areas/experiment.

### 4.8. Database and Statistical Analysis

The target genes and binding sites of the miRNAs were analyzed computationally using publicly available algorithms (TargetScan: www.targetscan.org, Pictar: pictar.mdc-berlin.de). Results are presented as the means ± standard errors of at least three independent experiments. Statistical significances were determined using the Student’s *t*-test for unpaired data.

## 5. Conclusions

In conclusion, this study reveals that miR-96-5p plays a crucial role during myogenic differentiation and myotube formation in C2C12 myoblasts. PA inhibited the expressions of myogenic factors and differentiation in myoblasts, and these inhibitions were accompanied by FHL1 reduction and miR-96-5p induction. Interestingly, miR-96-5p inversely regulated FHL1 expression by targeting the 3’UTR of FHL1 mRNA. Furthermore, the transfection of miR-96-5p mimic increased myoblast proliferation, reduced the expressions of myogenic factors, and impaired myogenic differentiation. Since there are no putative binding sites for miR-96-5p on the mRNAs of myogenic factors, we ascribe the inhibition of myogenic differentiation by miR-96-5p to direct FHL1 suppression in C2C12 myoblasts.

## Figures and Tables

**Table 1:** Primer lists and PCR conditions.

**(A) Mouse primer lists for *q*RT-PCR and RT-PCR**					
**Gene**	**Primer Sequence (5′-3′)**		**Product Size**	**Annealing Temp. (°C)**	**Cycle**
miR-96	F.P	TTTGGCACTAGCACATTTTTGCT	90	55	40 (*q*RT-PCR) 30 (RT-PCR)
	R.P	AGCAAAAATGTGCTAGTGCCAAA			
miRNA universal Primer	R.P	miScript universal primer (Qiagen)			
U6	F.P	CTCGCTTCGGCAGCACA	94		
	R.P	AACGCTTCACGAATTTGCGT			
FHL1	F.P	CTGAAGTGCTTTGACAAGTTC	102	58	
	R.P	GTGCCAGTAGCGATTCTTAT			
GAPDH	F.P	AACATCAAATGGGGTGAGGCC	252	58	
	R.P	GTTGTCATGGATGACCTTGGC			
CCND1	F.P	ACCAATCTCCTCAACGACCG	228	58	
	R.P	ACGGAAGGGAAGAGAAGGG			
CCNB1	F.P	GAGCTATCCTCATTGACTGG	125	58	
	R.P	CATCTTCTTGGGCACACAAC			
PCNA	F.P	GAACCTGCAGAGCATGGACTC	201	58	
	R.P	GGTGTCTGCATTATCTTCAGCCC			
**(B) Primer lists for the cloning of FHL1 3’UTRs**					
**3’UTRs**	**Primer Sequence (5′-3′)**		**Product Size**	**Annealing Temperature**	**Cycle**
wild-type FHL1 3’UTR	F.P	ATCTGGCCAACAAGCGCT	303	58	35
	R.P	AATTGCAGCCGGACAGAAA			
mutant FHL1 3’UTR	F.P	ATCTGGCCAACAAGCGCT	65		
	R.P	CTTTTTGCGTCAGTCGGGA			
	F.P	TCCCGACTGACGCAAAAAG	257		
	R.P	AAATTGCAGCCGGACAGAAA			

**Table 2:** Antibodies list.

Antibody	Manufacturer	Cat. No.
FHL1	Santa Cruz Biotechnology, Dallas, TX, USA	sc-374246
MyHC	DSHB, Iowa city, IA, USA	MF20
MyoD	Santa Cruz Biotechnology, Dallas, TX, USA	sc-377460
MyoG	Santa Cruz Biotechnology, Dallas, TX, USA	sc-12732
MEF2C	Thermo Fisher Scientific, Waltham, MA, USA	PA5-28247
β-actin	Sigma-Aldrich Chemicals, St. Louis, MO, USA	A2066
Antibodies HRP-linked anti-rabbit IgG	Cell Signaling Technology, Danvers, MA, USA	#7074
Goat anti-mouse(H + L)	Thermo Fisher Scientific, Waltham, MA, USA	#32430

All blots were visualized using a Femto reagent (Thermofisher Scientific).

## Supplementary Materials

The following are available online at https://www.mdpi.com/1422-0067/21/24/9445/s1.
