# Supplementary material for: MiR-96-5p Induced by Palmitic Acid Suppresses the Myogenic Differentiation of C2C12 Myoblasts by Targeting FHL1
Source: Int J Mol Sci. 2020 Dec 11;21(24):9445. doi: 10.3390/ijms21249445 (PMC7764195; doi:10.3390/ijms21249445)
Supplement: Supplementary file 1 [file ijms-21-09445-s001.pdf]

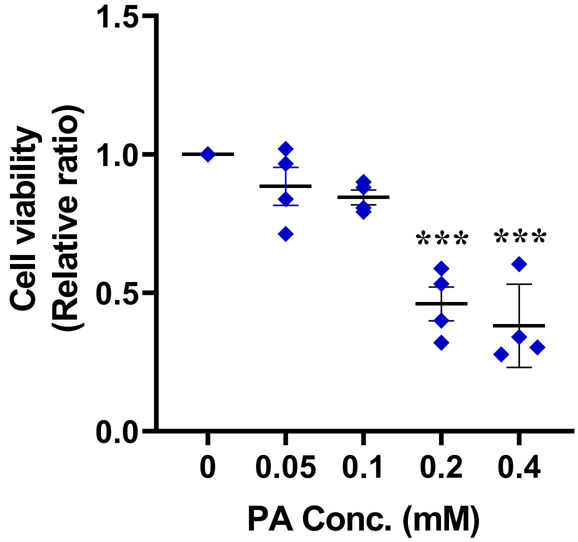

Figure S1. Cytotoxicity of PA in C2C12 myoblasts. Cytotoxicity at a given concentration of PA was assessed using a Quanti-Max Cell Viability Assay Kit (Biomax, Seoul, Korea) according to the manufacturer's instructions. Briefly, C2C12 cells were cultured in 96-well plates to expose to different concentrations of PA in triplicate for 24 h at 37°C. Next, 100 µl of media (DMEM) containing 10 µl Quanti-Max was added to each well. After 4 h incubation at 37°C, cytotoxicity was determined by using a microplate reader for absorbance measurements at 450 nm. Aligned dot plot are displayed as means ± SEMs (n = 4). ***, P<0.001 vs PA 0 mM.

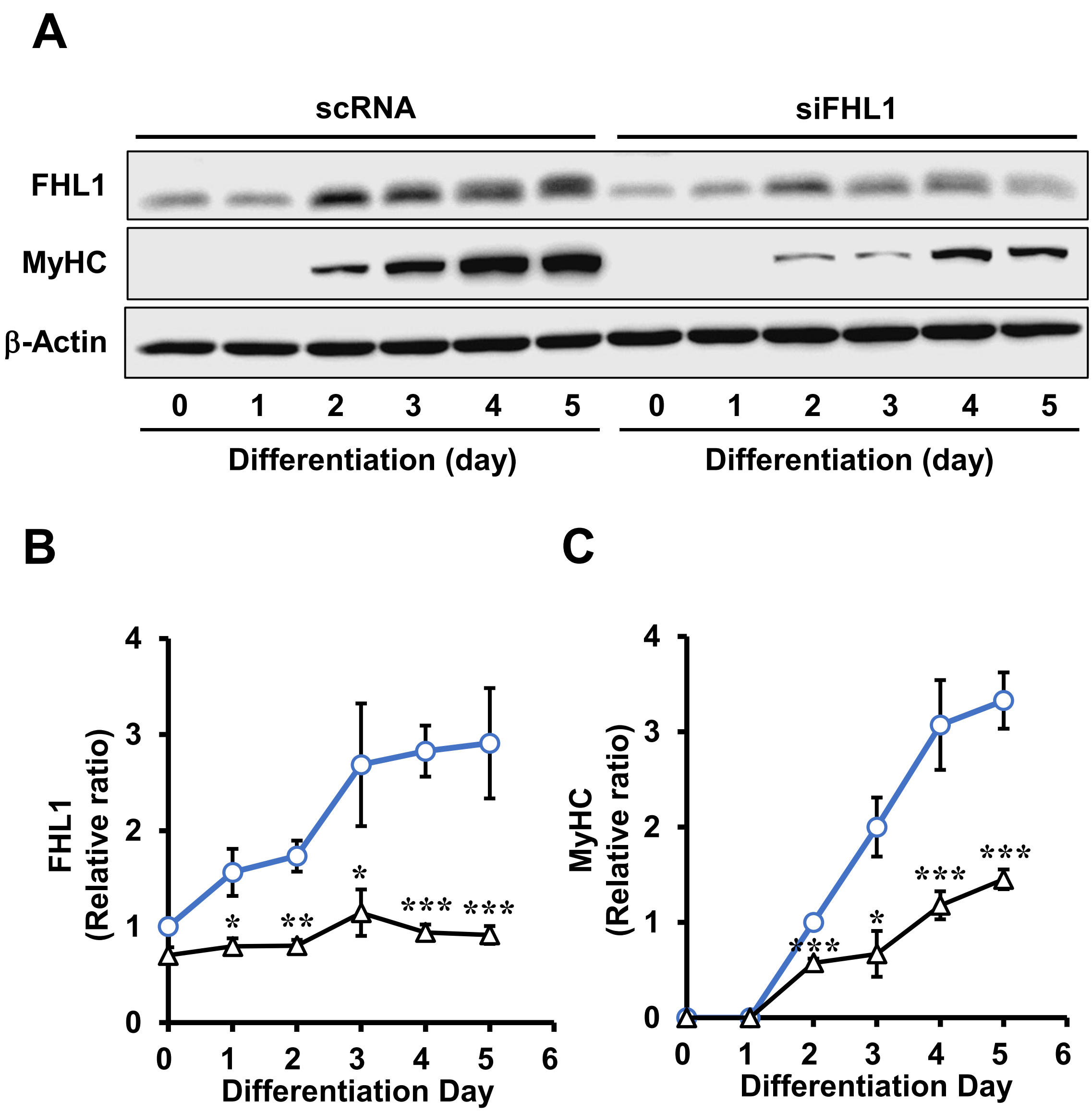

Figure S2. siRNA of FHL1 suppressed the expressions of FHL1 and MyHC. C2C12 myoblasts were transfected with 100 nM of scRNA control or FHL1 siRNA (siFHL1) and differentiated up to 5 days. (**A**) The expressions of FHL1, MyHC, and β-Actin were analyzed by immunoblotting. (**B**) Expression of FHL1 in immunoblots were determined by densitometry and normalized with β-Actin. (**C**) Expression of MyHC were measured by densitometry and were normalized with β-Actin. The values are expressed as the relative ratio of siFHL1 (triangle), where the intensity of normalized scRNA control (circle) was set to one. Values are expressed as means ± SEMs (n > 3). *, $P<0.05$; **, $P<0.01$; ***, $P<0.001$ vs scRNA.
